# Rich-Cores in Networks

**DOI:** 10.1371/journal.pone.0119678

**Published:** 2015-03-23

**Authors:** Athen Ma, Raúl J. Mondragón

**Affiliations:** Queen Mary University of London, School of Electronic Engineering and Computer Science, Mile End Road, London E1 4NS, United Kingdom; University of Gävle, SWEDEN

## Abstract

A core comprises of a group of central and densely connected nodes which governs the overall behaviour of a network. It is recognised as one of the key meso-scale structures in complex networks. Profiling this meso-scale structure currently relies on a limited number of methods which are often complex and parameter dependent or require a null model. As a result, scalability issues are likely to arise when dealing with very large networks together with the need for subjective adjustment of parameters. The notion of a *rich-club* describes nodes which are essentially the hub of a network, as they play a dominating role in structural and functional properties. The definition of a *rich-club* naturally emphasises high degree nodes and divides a network into two subgroups. Here, we develop a method to characterise a rich-core in networks by theoretically coupling the underlying principle of a rich-club with the escape time of a random walker. The method is fast, scalable to large networks and completely parameter free. In particular, we show that the evolution of the core in World Trade and *C. elegans* networks correspond to responses to historical events and key stages in their physical development, respectively.

## Introduction

Many networks exhibit a core/periphery structure which is important for the understanding of network properties and dynamics (for a review see ref. [1]). The constitution of such a structure often refers to a simple two-class partition [2–4], and a core is said to be comprised of densely inter-connected high-degree nodes which govern flows and impact adaptability, flexibility, and controllability in networks [5, 6]. The definition of core/periphery was formalised in [2] and numerous profiling methods developed were based on optimising a suitable fitness function, such as using a coreness value to define the density of links inside the core [2]; referring to a quality index with respect to the size of the expected core and the fuzziness of the boundary [7]; or applying Markov chains to describe random walks so as to index the coreness of individual nodes [8]. These methods rely on subjective fine-tuning due to the presence of one or more *free parameters* which are often obtained arbitrarily or by techniques such as simulated-annealing. Other examples include maximising the closeness centrality within the core by using an ensemble of random networks to define a coefficient that characterises the core [3], and this imposes a requirement of a statistical null model. Generally, these methods tend to be relatively complex in nature and therefore scalability issues are likely to be encountered when applied to very large networks.

The notion of a rich–club is used to describe the connectivity between high degree nodes, and it has been applied to profile meso–scale properties in networks by examining the density of connections between high degree nodes [9–14]. A rich–club influences the functionality of a network, as demonstrated in the transmission of rumours in social networks [15], the delivery of information in the Internet [9], its strong effect on both the network assortativity and transitivity [11], and the organisation of the human connectome in neurodevelopment [16, 17]. Interestingly, the presence of a rich–club naturally divides a network into two parts; this means that the definition of a rich–club coincides with two fundamental prerequisites of a core/periphery structure: *high degree nodes* and a *two-class partition*. However, there is, at present, no general method to define which high degree nodes are members of the club [11, 13, 18].

Here, we present a *rich–core* method which profiles the core/periphery structure. The method brings together the concept of a rich–club and the diffusion of a random walker and defines a rich–core by examining the persistence probability of random walks among high degree nodes. We apply the method to a wide range of networks, ranging from man-made, social and biological, with a variety of network sizes. Furthermore, our results show that the core closely reflects the development and re–alignments of relationships over time in evolving networks. For example, changes in the core coincide with timing of historical events in trade development and with key developmental stages in a biological process. Anomalous nodes in the core can be uncovered with reference to null models by discriminating statistical differences. In addition, as the method does not levy any restriction on connectivity, the core can exhibit different properties, such as a core with an internal structure or a multi-core. The rich–core method is simple, fast, entirely free of any external parameters and applicable to very large networks, providing an effective and yet generic way to examine meso-scale properties in complex networks.

## Results

Consider an unweighted and undirected graph. We rank the importance of the nodes in descending order of their degree, such that the node with the highest degree is ranked first and so on, and nodes are then re-labelled according to their rank. For a given node, we divide its links into two groups: those with nodes of a higher rank and those with a lower rank. More formally, a node with a rank *r* has degree *k*_*r*_; the number of links it shares with nodes of a higher rank is $k_{r}^{+}$ and the number of links with nodes of a lower rank is $k_{r}-k_{r}^{+}$. Core nodes are high degree nodes that are densely connected with each other [2], and we assume that the connectivity of a highly ranked node with other *higher* ranked nodes contributes towards the constitution of a core. Similarly, if a node has very few links with higher ranked nodes, it is likely to be a member of the periphery. To detect a core we propose this straightforward procedure (see Materials and Methods for the full technical description). Starting from the node with the highest rank, as *r* increases the number of links $k_{r}^{+}$ that node *r* shares with nodes of a higher rank fluctuates. There will be a node r* where $k_{r}^{+}$ has reached its maximum, and from that node onwards $k_{r}^{+}$ is always less than $k_{r^{*}}^{+}$. This change in the connectivity among the highly ranked nodes defines the boundary of a (rich) core; the nodes with a rank less than or equal to r* are the core and the rest belong to the periphery.

**Fig 1:**
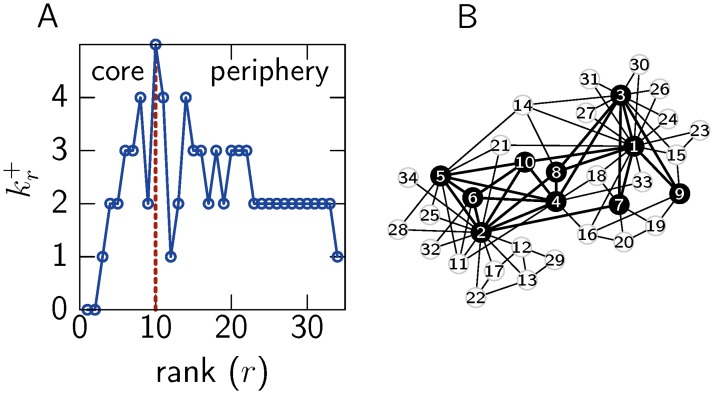
The rich–core for the Zachary Karate Club network. (A) The number of links $k_{r}^{+}$ that node *r* shares with nodes of a higher rank. The boundary of the core is marked by $k_{r^{*}}^{+}$ where $k_{r}^{+}$ is at its maximum, as indicated by the dotted line. (B) A graphical representation of the network with core (black) and periphery (white) nodes derived from the rich–core method.

This pragmatic way of defining a core is related to the concept of a random walker in a network [8]. Consider a network which is partitioned into two sets: *S*_*c*_ is the core and *S*_*p*_ is the periphery. A random walker jumps from one node to another following a link between any pair of nodes, and the probability of the walker to visit a given node is proportional to the node’s degree. The time it takes a random walker to escape from *S*_*c*_ to *S*_*p*_ is *τ*_*c*_. If *S*_*c*_ is the set of nodes with the highest rank, as we include another node into this set the escape time will naturally increase. Eventually, *S*_*c*_ will contain all the nodes in the network, and the escape time will converge as the random walker is always contained in this set and has nowhere to escape to. This means that if we begin by putting only the top ranked node in set *S*_*c*_, and gradually increase the size of *S*_*c*_ by adding nodes in decreasing order of their rank, the escape time will always increase. If we consider the rate of change of the escape time as nodes are added to the set, the boundary of a rich–core is defined as the point in which the rate of increase in the escape time changes from a slow to a fast pace. This point coincides with the rank r* defined above (as shown in Materials and Methods) which establishes a direct relationship between the cohesiveness of a core reflected by the escape time of a random walker and the boundary of a rich–core. Hence, the method itself does not impose any external parameter when defining a core. We examine the Zachary Karate Club network [19] which describes the friendships among members of the club. Fig. 1A shows that nodes are ranked in descending order of degree and the way in which $k_{r}^{+}$ changes as additional nodes are included. The rich–core of the network is bounded by the maximum $k_{r}^{+}$ and is formed by the 10 highest ranked nodes as shown in Fig. 1B, including instructor Mr Hi and President John A., represented by node 1 and 2 respectively, who disagreed on the issue of lesson fees and led to a split in the club, and a number of their high degree followers.

### Relative core size

**Fig 2:**
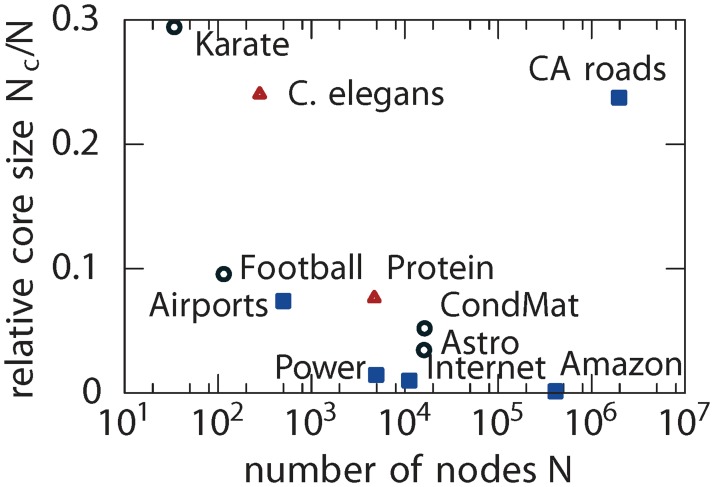
Relative size of the core in different kinds of real networks. Man-made (square): US airports (Airports) [22], Amazon.com recommendation (Amazon) [23], Californian (CA) roads [21], Internet [24] and Power grid (Power) [20]. Social (circle): Astrophysics collaborations (Astro) [25], Condensed Matter collaborations (CondMat) [25], American College football (Football) [26] and the Zachary Karate club (Karate) [19]. Biological (triangle): *C. elegans* [20] and Protein [27].

The size of a core is an important property of a network as it has been suggested that a sizeable core makes a network more flexible and adaptable to changes [5], and a small core makes a network more controllable [6]. Here, we study the relative core size, *c*, which is the ratio between the number of nodes in the core *N*_*c*_ relative to the total number of nodes in the network *N*, across a wide range of networks. A network that has no periphery will have a relative core of 1, e.g. a fully connected network; a star network with *N* nodes has a relative core of 1/*N* and a core-less network has no links. Fig. 2 shows the cores observed in networks which are different in size and structure (see Table A in S1 File for details), in part reflecting their functionality. For instance, the Amazon.com recommendation network and the Internet both have a relatively small core. The former is found to be disjoint and, as the network contains information about product recommendations, the results provide evidence of efficient information transfer within the network but only restricted to localised parts. The latter has a well connected single core which reflects its design for efficient routing between Autonomous System domains. The *C. elegans* neuronal network [20] has a relatively large single core, and this perhaps reflects the adaptability of the neuronal network to living conditions. The Californian road network [21] has a relatively large disjoint core which represents the existence of many crossroads, providing great flexibility in route choices as they present many possibilities between different geographical points. We did not observe any characteristic size of the core related to the origin of networks, i.e. man-made, social or biological. We compare our results with those obtained from using the core profiling (CP) method in [8] (Table A in S1 File). While the two methods identify many common high degree nodes in the core, the latter is more likely to define much larger cores by incorporating relatively low degree nodes (Figure A in S1 File) that do not necessarily fall under the formal definition of a core node [2].

### Weighted and directed networks

The definition of a rich–core can firstly be extended to weighted networks [28, 29]. Consider *w*_*min*_ is the minimal weight linking two nodes in a network and the link between nodes *i* and *j* has a weight of *w*_*ij*_. This link is represented by ⌈*w*_*ij*_/*w*_*min*_⌉ links and the ranking is performed in units of the minimal weight. Each node is assigned to *σ*_*i*_ = ∑_*j*_⌈*w*_*ij*_/*w*_*min*_⌉ links, and part of this quantity arisen from the node’s linkage to nodes of a higher rank is referred to as $\sigma_{r}^{+}$; similarly, the remaining proportion, $\sigma_{r}-\sigma_{r}^{+}$, is the normalised weight that node *r* shares with nodes of a lower rank. The core boundary is node r* such that $\sigma_{r^{*}}^{+}>\sigma_{r}^{+}$ for *r* > r*.

Similarly, the notion of a rich–core can equally be applied to directed networks by dividing links into in– and out– links and quantifying their corresponding weights. In this case the definition of a rich–core does not only depend on the weight of the nodes but also the direction of their links. An example is the assessment of web pages using PageRank where it has been observed that the popularity of a node is closely related to its in–degree [30]. Here, the direction of interest defines the in–links which determine the ranking (see Materials and Methods). Let $\sigma_{r}^{+in}+\sigma_{r}^{+out}$ be the total strength of interactions between node *r* and the nodes *r*′ < *r*, as both in– and out– links contribute towards the cohesiveness of a core, and the core boundary is the node r* such that $\sigma_{r^{*}}^{+in}+\sigma_{r^{*}}^{+out}>\sigma_{r}^{+in}+\sigma_{r}^{+out}$ for *r* > r*.

**Fig 3:**
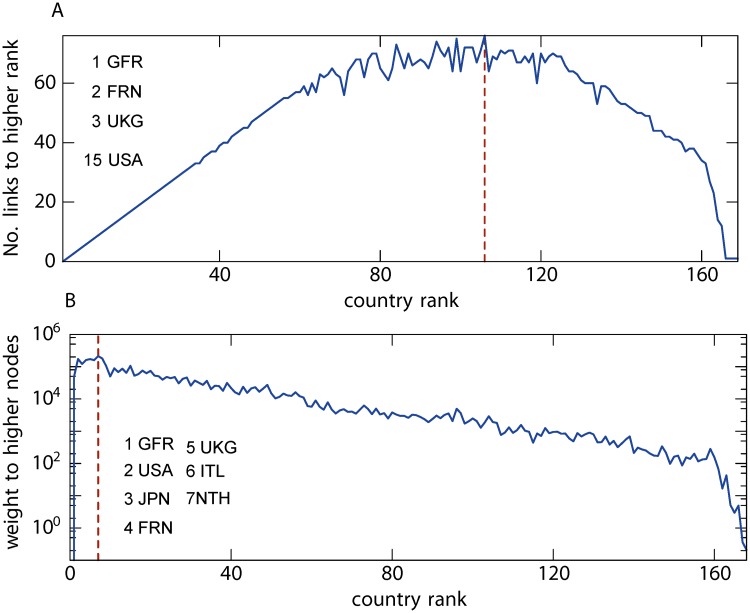
The World Trade network in 1990. (A) The unweighted undirected network which represents trade relationships between the countries. The core has 106 countries. (B) The weighted directed network representing the exchange of wealth between the countries. The 7 countries in core are listed.

We refer to the World Trade network [31] as both an unweighted and weighted (directed) network whereby nodes are countries and links are trade channels; the latter can represent the direction of trade to specify an import or export relationship in a directed graph. The associated financial value can be seen as the weight of a given link. The overall connectivity of the network is found to be high as countries are interrelated in many ways. We first examine the network as *an undirected and unweighted* network and a link simply refers to the presence of trade. In 1990, a total of 60% of all the countries in the world were part of this network as a result of globalisation of trading at the time [32]. By ranking countries in descending order of their degree, Fig. 3A shows the number of trade relationships each of these countries has with countries of a higher rank. There are 106 countries in the core, which is similar in size to previous findings [8, 33]. The highest ranked nodes in the core (Germany, France, the UK) confirmed close trading relationships found among the countries within the European Union (EU) and the rest of the world. They were closely followed by other long established countries in the EU (e.g. Italy and the Netherlands) and the USA. Historically, these countries have well established trade with many other countries. The core provides an indication of the magnitude of interlinkage among developed countries and countries with strong manufacturing or agriculture (e.g. Brazil and Kenya); and countries outside the core are mostly confined to developing countries in Africa or countries that are very small in physical size (e.g. Andorra and San Marino). Now, if we take both the direction and weight into consideration, and nodes are ranked in the descending order of their export which is interpreted as the generated income towards the country of origin, defining the weighted in-degree, and Fig. 3B shows that there are 7 members found in the core and they are consistent with the top exporters in the world at the time (in 1990) [33]. Coincidently, these countries were also the top importers as there were high degrees of symmetry in financial values in the two trading directions [32].

### Evolution

**Fig 4:**
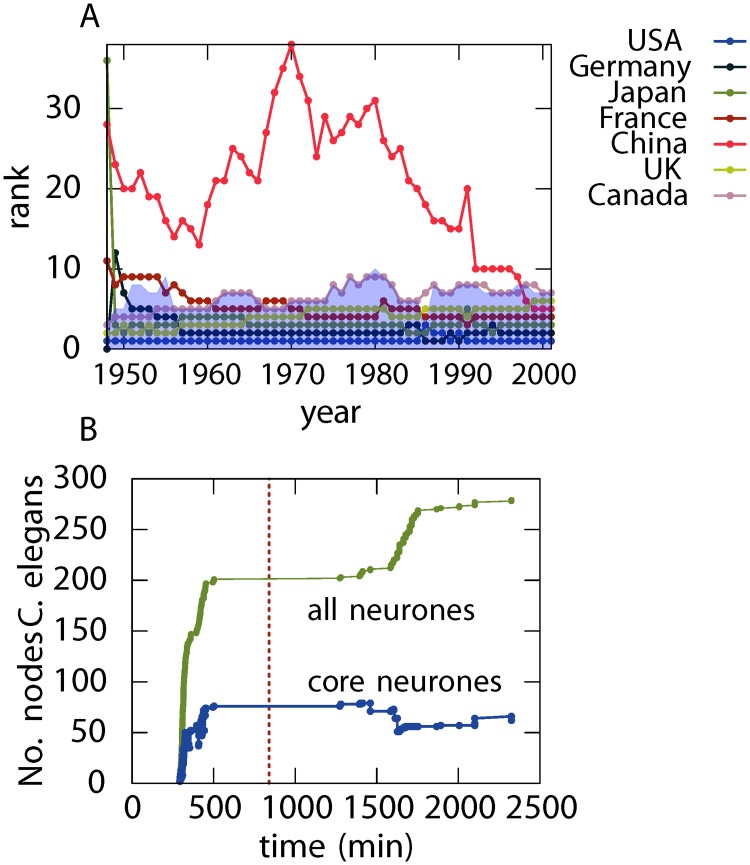
Evolution of the core of the World Trade and the *C. elegans* networks. (A) The World Trade network in which the shaded area in the background is the core region and countries in the core in a given year lie within this area. The countries shown are the members of the core in year 2000, and the individual lines are their ranks in a given year. The USA, Germany, Japan, France and the UK have already been found in the core, though there are small variations in the rank over the years. Canada is mostly found at the edge of the core during the period shown. China lies relatively far away from the core prior to the 1990s and it becomes part of the core in 1997. (B) Evolution of the core size for *C. elegans*. The numbers of nodes in the core and in the neural network are plotted against development time and the red dotted line marks the hatching time.

Networks are often found to be temporal in nature as they are subject to formation, dissolution and rewiring of links [34, 35]. Continuing with the example of World Trade we examine the evolution of the core between 1948 to 2000. While the number of participating countries continued to grow over time, the core of the directed and weighted network consists of a very selective group, corresponding to 4% to 6% of all the countries (S1 Fig.). This can be explained by referring to the way in which World Trade has grown since the Second World War. International trading is said to be growing steadily but unevenly since the 1940s, as trade barriers were imposed by events such as the Cold War. The network was also strongly influenced by other key historical events, geographical distance, composition (e.g. products and services) and the nature of trade [32]. Throughout the 1980s and 1990s, there was a substantial reduction in the cost of shipping due to the explosion of air freight, the collapse of the Soviet Union leading to many independent countries, and the industrialisation of developing countries; all these events has shaped the development of trade worldwide, leading to a great leap in globalisation. This is closely reflected by the way the membership of the core has changed over time in Fig. 4A. The USA, Germany, Japan, France and the United Kingdom were the top importers and exporters in the world during the period of study and it can be seen that these countries have been members of the core during the entire time. Canada was drifting in and out of the top ten in the World Trade ranking during the same period, and we can see a similar variability in its core membership. In addition, the economic reform in China, which started in the late 1970s, has led to a steady growth of ∼ 9% in World Trade per year, and our results illustrate that China became a member of the core in 1997 which is just before China joining the World Trade Organisation in 2001.

Another example of how changes in the core tie in with key events in real networks can be found in the physical development of *C. elegans* neuronal connections in Fig. 4B. The core of a fully developed worm contains 61 (S1 Table) out of a total of 302 neurones, and almost the entire core is developed within the first 500 minutes [13]. The formation of the core coincides with Embryogenesis, and it has been suggested that the highly connected neurones appear in the early development so as to minimise the energy cost by creating the core connections among key nodes that are not physically far apart [36, 37]; these connections can then be extended during the process of body elongation. New neurones are found after hatching in the late L1 larval stage at approximately 1250 minutes and the total number of neurones continues to grow until the start of the L4 larval stage at approximately 2400 minutes [38]. The post-hatching development causes the relative connectivity among the existing core neurones to decrease, resulting in a reduction in the overall size of the core, from its maximum size of 79 neurones at 1459 minutes to 61 neurones. The shrinking of the core coincides with the timing of Gonadogenesis.

### Properties of the core

**Fig 5:**
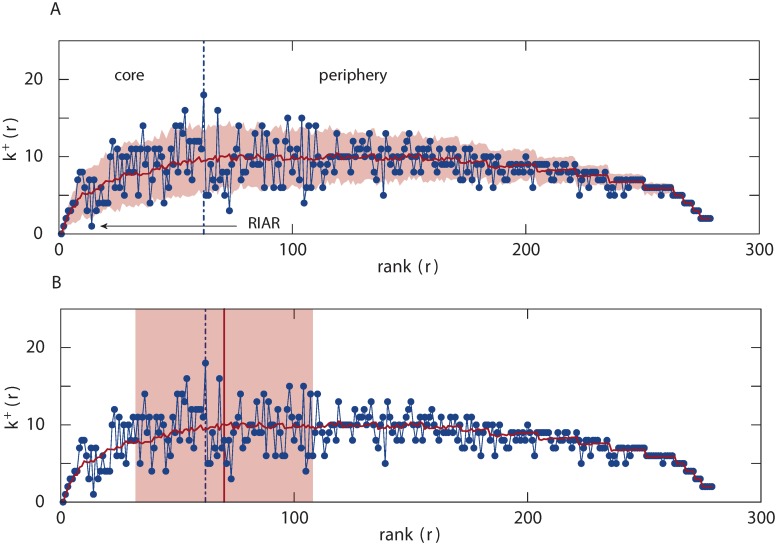
Comparison on the connectivity of the core between the empirical data and the null model for *C. elegans*. (A) The shaded area marks two standard deviations from the sample mean. Neurone RIAR is found to have an anomalous connectivity with the highest ranked nodes. (B) Comparison on the core size between the empirical data (blue line) and the null model (red line). Similarly, the shaded area shows two standard deviations.

Certain properties of a core can be revealed by examining how the value of $k_{r}^{+}$ changes as additional nodes are included. For example, Fig. 5A shows $k_{r}^{+}$ plotted against *r* for the *C. elegans* network, and it can be seen that the top thirteen neurones are well connected, forming a tight cluster but sharing only one link with neurone RIAR which is ranked 14. Previously, only the top 14 nodes have been identified as the rich–club of the network [13]. Here, we show that the core is comprised of a set of closely connected high degree nodes interlinking with another set of high degree nodes immediately after neurone RIAR until the boundary of the core is reached. While the method itself does not require any null model to define a core, comparisons with a null counterpart, however, do provide a way to detect any anomalies with respect to the core size and connectivity among its members. We employ a randomisation method to create an ensemble of networks which in turn are used as a reference null model for comparison purposes. The randomisation is restricted to preserve the ranking of the nodes, that is the weight/degree of the nodes (Materials and Methods). Continuing with the example of the *C. elegans* network, we create an ensemble of 100 networks with the same degree distribution as the original network. From the ensemble we evaluate the average number of links that node *r* has with nodes of a higher rank, i.e. $\left\langle k_{r}^{+}\right\rangle$, and the standard deviation of this quantity. Fig. 5A also shows the number of links between a node of rank *r* and a node of rank *r*′ < *r* and the average number of links obtained from the null–model. The boundary of the core/periphery is the dotted blue line. The red line is $\left\langle k_{r}^{+}\right\rangle$ and the pink shaded area demarcates two standard deviations from the mean value (numerically verified as the 95th percentile). This implies that nodes outside the shaded area can be considered anomalous. The fact that neurone RIAR shares only one link with nodes of a higher rank has been highlighted here, suggesting there is an anomaly in the connectivity. The randomisation can also be used to decide if the size of a core is within the expected value. Fig. 5B shows the average size of the core obtained from the ensemble of networks (red dotted line) and the pink area corresponds to two standard deviations from this mean. The dotted blue line is the size of the core obtained from the empirical data; as this value falls inside the pink area we can conclude that the size of the core for the *C. elegans* is what we expected.

## Discussion

We develop a method to profile core/periphery in networks by coupling the notion of a rich–club and the persistence probability of random walks, resulting in the definition of a rich–core. In contrast to existing techniques that depend on one or more free parameters [2, 7, 8] or a null reference [3], our method only refers to the degree sequence and requires no prior knowledge on the network. As a result, the method is simple, fast and scalable, and particularly advantageous for profiling very large networks; as shown in the Amazon.com recommendation and Californian Road networks in which the method in [8] was found to be too computationally costly to run (Table A in S1 File). Our method is generic and is applicable to a wide range of networks as demonstrated by the results, and by examining the relative core size it is possible to gain an insight into the flexibility and controllability [5, 6] of a variety of networks. Though, it is worth noting that approximating the persistence probability by a continuous function when profiling a core may lead to a small degree of deviation from the actual boundary, and this would particularly be the case among networks without a sharp core and periphery transition [7]. Similarly, degeneracy in the ordering of nodes originating from nodes with the same degree may cause the boundary to shift. In both cases, the impact on the actual core is found to be minimal.

Furthermore, we uncover characteristics of real networks through the profiling of the core/periphery structure, contributing towards the understanding of the significance of such meso-scale property. In particular, development and realignment of relationships are often found in networks with temporal nature. The core identified in the World Trade network represents the extent of trading among the developed countries and countries with a strong presence of manufacturing or agriculture, and the evolution of the core reflects closely how key historical events have shaped the patterns of import and export. Our results have demonstrated that the world’s dominance in trading not only conformed to long-established countries but was also joined by emergent countries such as China in recent decades. Similarly, the *C. elegans* network shows that temporal changes of the core closely tie in with the key stages in the species’ physical development. The proposed method does not impose any conditions on the connectivity inside the core and provides an effective way to reveal any internal organisation of a core, such as a multi-core. We show an effective way to identify any anomalies in the core by comparing the membership of the core of a given network with its null counterparts, and how this approach can be used to evaluate the expected size of of a given core.

Compartmentalisation in networks has been suggested to be a key factor in facilitating network robustness [39]. A core organisation in networks is associated with structural properties such as symmetry, assortativity and hierarchy [1], and can be seen as an integrative function that provides redundancy for fluxes. However, we have yet to fully assess the impact of a core on network fragility, and the related dynamical properties are largely unexplored. A better understanding on these aspects will gain an insight into the functional role of a core and its impact on the overall network behaviour. We envision that the scope for cross-fertilisation here is enormous as networks, particularly those that are biological in nature, are often comprised of functional groups [40] and factors that may contribute towards their stability are currently still under debate.

## Materials and Methods

### Rich–club Coefficient and Core/Periphery Profile

To find the boundary of a rich–core, we examine the escape time it takes a random walker to leave a core. The escape time is related to the notion of persistence probability *α* which indicates the cohesiveness in a subgraph [8, 41]. The persistence probability of cluster *S*_*c*_ is

$$\begin{array}{c} \alpha_{c}=\frac{\sum_{i,j\in S_{c}}\pi_{i}m_{ij}}{\sum_{i\in S_{c}}\pi_{i}}. \end{array}$$ (1)

where *π*_*i*_ is the probability that a random walker is in node *i*, and *m*_*ij*_ is the probability that a random walker moves from node *i* to node *j*. The escape time is *τ*_*c*_ = (1−*α*_*c*_)^−1^. Assuming that *α*_*x*_ can be approximated by a continuous function *α*(*x*) = *g*(*x*)/*f*(*x*) where *x* is a continuous rank, the proposed method seeks to find the boundary of the core by locating the point at which the transition from a low to a high persistence probability accelerates. The function *α*(*x*) increases with *x* as the number of nodes in the cluster rises, and eventually converges to 1 when all the nodes are included. Therefore, the first derivative of *α*(*x*) is always positive and the value of *x* for which the rate of increase is maximal is obtained when the second derivative is zero, i.e. *α* ^″^(*x*) = 0, where *α* ^″^(*x*) = *g* ^″^(*x*)/*f*(*x*)+2*g*(*x*)*f*′(*x*)^2^/*f*(*x*)^3^−2*f*′(*x*)*g*′(*x*)/*f*(*x*)^2^−*g*(*x*)*f* ^″^(*x*)/*f*(*x*)^2^. To first approximation *α* ^″^(x*) ≃ 0 if *g* ^″^(x*) = 0 as *f*(*x*) is a positive increasing function of *x*.

For undirected networks, *α*_*c*_ is given by the sum of the number of links between the nodes in *S*_*c*_ divided by the sum of the degrees of the nodes in *S*_*c*_. If *a*_*ij*_ are the elements of the adjacency matrix, then

$$\begin{array}{c} \alpha_{c}=\left(\sum_{i,j\in S_{c}}a_{i,j}\right)/\sum_{i\in S_{c}}k_{i}=2\frac{\sum_{i=1}^{c}k_{i}^{+}}{\sum_{i=1}^{c}k_{i}}. \end{array}$$ (2)

Again, assuming that *α*_*c*_ can be approximated with a continuous function *α*(*x*) = *g*(*x*)/*f*(*x*) where $g(x)=\int_{1}^{x}k^{+}(y)dy$ then *g* ^″^(x*) = 0 means that *k* ^+^(x*) has a maximum or a minimum at the value x*, in this case we are interested in the maximum. We refer to the point x* where *g* ^″^(x*) = 0 as the boundary of the rich–core and nodes in *S*_x*_ are the members of the core.

The rich–club coefficient measures the density of links among (high degree) nodes and is defined as [9]:

$$\begin{array}{c} \phi \left(r\right)=\frac{2E(r)}{r(r-1)}=\frac{2\sum_{i=1}^{r}k_{i}^{+}}{r(r-1)} \end{array}$$ (3)

where *E*(*r*) is the number of links between the *r* nodes. The explicit relationship between the escape time and the rich–club coefficient is obtained by substituting Eq. (3) into Eq. (2), giving

$$\begin{array}{c} \alpha_{r}=\frac{r(r-1)\phi (r)}{\sum_{i=1}^{r}k_{i}}. \end{array}$$ (4)

For weighted networks, as we are considering the weights as undirected multilinks, the same argument applies when defining a core. For directed networks, the persistence probability, *α*_*c*_, is given by the ratio between the number of times a random walker transits inside the core and the number of times it visits the core. The former is proportional to the total number of links inside the core, which we denoted as $\sigma_{r^{*}}^{+in}+\sigma_{r^{*}}^{+out}$, as both in– and out– links provide paths for the random walker to move *within* core and hence contribute towards the persistence probability. The latter is given by the sum ∑_*i* = *S*_*c*__ *π*_*i*_. The in–degree *k*_*i*_ of node *i* is assumed to be a good approximation of *π*_*i*_ [30] which is characterised by the direction of interest (as demonstrated in the example of World Trade); hence, the number of times a random walker visits the core increases with *r*, as the nodes are ranked in decreasing order of their in–degree.

To find the core of a given network,

1. rank nodes in decreasing order of their weight (specifically, degree, in–degree and weight for undirected, directed and weighted networks respectively).
2. evaluate the number of links *k* ^+^ between node with rank *r* and nodes with rank *r*′ < *r*
3. find the boundary of the core, defined by the node r* where $k_{r^{*}}^{+}>k_{r}^{+}$ for all *r* > r*

### Rank degeneracy

As numerous nodes can have the same degree, the ranking of nodes with equal degree is not strictly defined. This degeneracy in the ranking scheme would affect the determination of the boundary nodes, and hence the size of a core. To evaluate the effect of the degeneracy in the definition of a core we randomly re-rank the nodes with equal degree and measure the change of the core nodes. We observe that this re–ranking only has a minor effect when defining a core.

### Construction of Null Models

The null model is generated using the Zlatic *et al.* approach [29] which is a generalisation of Maslov, Sneppen and Zaliznyak method (MSZ) [42] to generate null models. Zlatic *et al.* redistributes the weights of the links by preserving the strength of the nodes as follows. If *w*_*min*_ is the minimum value of the weights in the original network then the rewiring is done by changing the weights of two pairs of links by an amount *w*_*min*_. The rewiring consists of selecting two links at random and exchanging one of the end nodes of the first link with an end node of the second link.

## Supporting Information

S1 File Comparisons between the core profiling (CP) and rich-core (RC) methods. (PDF)

S1 Fig The relative core size of the directed and weighted World Trade network between 1948 and 2000. (PDF)

S1 Table Neurones that form the rich–core of the *C. elegans*. Rank, degree and birth time for all core neurones are shown.
(PDF)
