# Supplementary material for: Rich-Cores in Networks
Source: PLoS One. 2015 Mar 23;10(3):e0119678. doi: 10.1371/journal.pone.0119678 (PMC4370710; doi:10.1371/journal.pone.0119678)
Supplement: S1 File — (PDF) [file pone.0119678.s001.pdf]

S1 Supplementary Information

Rich-cores in networks

Athen Ma & Raúl J. Mondragón

Queen Mary University of London
School of Electronic Engineering and Computer Science
Mile End Road, London E1 4NS United Kingdom

We refer to the core profiling (CP) method proposed by Della Rossa *et al.* [1] for comparison. Generally, the cores obtained by the CP method are much larger than their rich-core (RC) counterparts, though most of the rich-core nodes are also identified by the CP method (see column "Common nodes" in Table A). In Figure A, nodes are in descending order of degree and plotted against their degree, and core nodes obtained from the CP and RC methods are denoted in green triangles and red circles respectively. We see that the additional nodes identified in the CP cores are mostly nodes with relatively low degrees. For example, in the Karate network there are nodes in the CP core with a degree $k = 3$. see Figure A(i). In the examples of the Amazon.com recommendation and Californian road networks, we are unable to compute the core using the CP method due to their size ‡.

Table A. Comparisons between the core profiling (CP) and rich-core (RC) methods. Properties related to the individual networks are shown, together with the actual and relative size of the cores obtained via the two methods.

Network	Nodes	Links	Degree distribution	Nodes in core CP	Nodes in core RC	Common nodes	Relative size CP	Relative size RC
Karate [2]	34	78	†	14	10	9	0.412	0.294
Football [3]	115	613	†	97	105	92	0.843	0.913
World Trade 1990 [4]	169	7991	Log-normal	132	106	105	0.781	0.627
C. elegans [5]	279	6264	Power-law tail	200	62	61	0.717	0.222
Airports [6]	500	2980	Power-law	203	39	39	0.406	0.078
Protein [7]	4713	14846	Power-law	1896	181	180	0.402	0.038
Power [5]	4941	6594	Exponential	2281	60	58	0.462	0.012
Internet [8]	11174	23409	Power-law	1673	110	110	0.150	0.010
Astro [9]	16046	121251	Power-law	10244	565	565	0.638	0.035
CondMat [9]	16264	47594	Power-law	9444	875	874	0.581	0.054
Amazon(.com) [10]	410236	2439437	Power-law	n/a	567	n/a	n/a	0.001
Californian [11]	1965206	5533214	Exponential	n/a	468148	n/a	n/a	0.238

† The network is too small in size to determine the exact distribution.
‡ Both CP and RC methods were run in Matlab, using 200GB of RAM on the capacity cluster, Apocrita, hosted at the authors' university which consists of 1800 Westmere cores (150 nodes) with 24GB of RAM, plus 11 nodes with 48 cores and 512 GB of RAM.

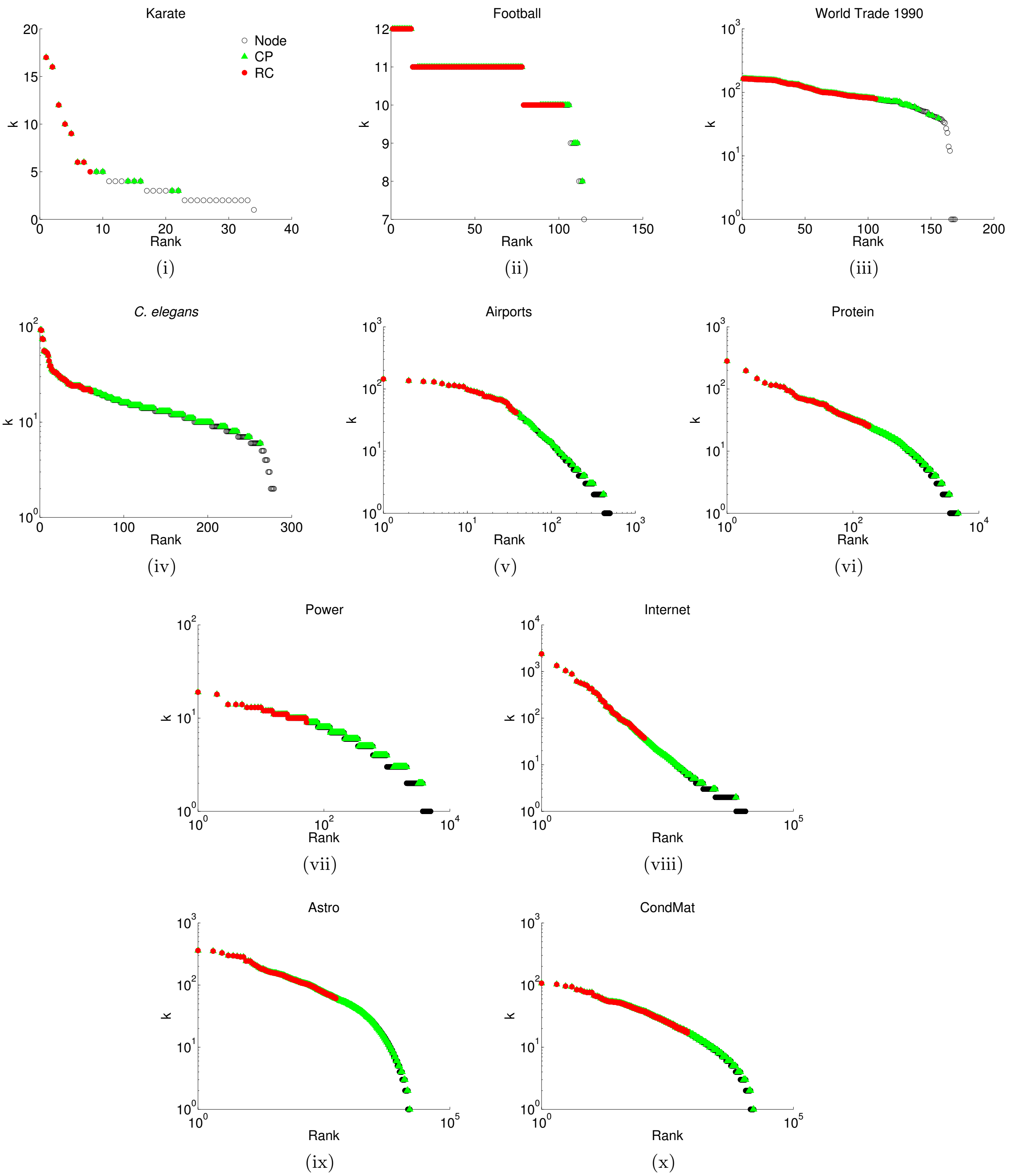

Figure A. Comparisons on the distributions of core nodes obtained by the core profiling (CP) and rich-core (RC) methods.

References

[1] Della Rossa F, Dercole F, Piccardi C. Profiling core-periphery network structure by random walkers. Scientific reports. 2013;3.

[2] Zachary WW. An information flow model for conflict and fission in small groups. Journal of anthropological research. 1977;p. 452–473.

[3] Girvan M, Newman ME. Community structure in social and biological networks. Proceedings of the National Academy of Sciences. 2002;99(12):7821–7826.

[4] Gleditsch KS. Expanded trade and GDP data. Journal of Conflict Resolution. 2002;46(5):712–724.

[5] Watts DJ, Strogatz SH. Collective dynamics of small-worldnetworks. Nature. 1998;393(6684):440–442.

[6] Colizza V, Pastor-Satorras R, Vespignani A. Reaction–diffusion processes and metapopulation models in heterogeneous networks. Nature Physics. 2007;3(4):276–282.

[7] Jeong H, Mason SP, Barabási AL, Oltvai ZN. Lethality and centrality in protein networks. Nature. 2001;411(6833):41–42.

[8] Chen Q, Chang H, Govindan R, Jamin S. The origin of power laws in Internet topologies revisited. In: INFOCOM 2002. Twenty-First Annual Joint Conference of the IEEE Computer and Communications Societies. Proceedings. IEEE. vol. 2. IEEE; 2002. p. 608–617.

[9] Newman ME. The structure of scientific collaboration networks. Proceedings of the National Academy of Sciences. 2001;98(2):404–409.

[10] Leskovec J, Adamic LA, Huberman BA. The dynamics of viral marketing. ACM Transactions on the Web (TWEB). 2007;1(1):5.

[11] Leskovec J, Lang KJ, Dasgupta A, Mahoney MW. Community structure in large networks: Natural cluster sizes and the absence of large well-defined clusters. Internet Mathematics. 2009;6(1):29–123.
