# Supplementary figures and images for: Rich-Cores in Networks
Source: PLoS One. 2015 Mar 23;10(3):e0119678. doi: 10.1371/journal.pone.0119678 (PMC4370710; doi:10.1371/journal.pone.0119678)

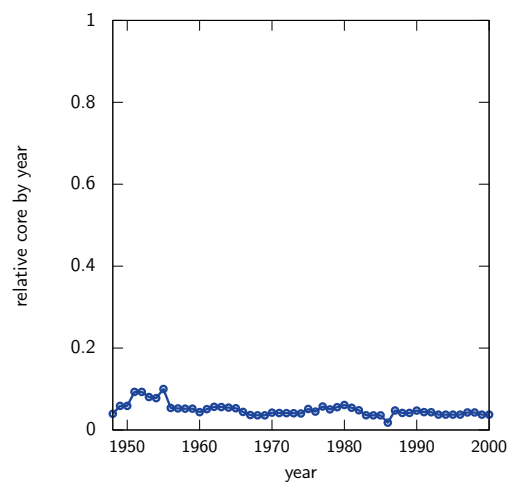

**Figure S1.** The relative core size of the directed and weighted World Trade network between 1948 to 2000.

Supplement: S1 Fig — (PDF) [file pone.0119678.s002.pdf]
