# Supplementary material for: Rich-Cores in Networks
Source: PLoS One. 2015 Mar 23;10(3):e0119678. doi: 10.1371/journal.pone.0119678 (PMC4370710; doi:10.1371/journal.pone.0119678)
Supplement: S1 Table — Rank, degree and birth time for all core neurones are shown. (PDF) [file pone.0119678.s003.pdf]

Table S1. Neurones that form the rich–core of the *C. elegans*. Rank, degree and birth time for all core neurones are shown.

Rank	Neurone	Degree	Birth time
1	AVAR	93	311.2875
2	AVAL	92	312.2642
3	AVBL	75	318.4751
4	AVBR	74	314.0625
5	AVER	56	328.7478
6	AVEL	55	325.4717
7	AVDR	55	300.7055
8	PVCL	54	448.6804
9	PVCR	53	450
10	DVA	50	296.25
11	AVDL	44	298.9418
12	AIBR	39	306.1728
13	RIBL	38	307.0381
14	RIAR	36	326.2787
15	AVKL	34	301.7595
16	RIBR	34	315.9375
17	AIBL	34	295
18	RIGL	33	321.9941
19	RIAL	33	332.4515
20	HSNR	33	411.9929
21	RIH	32	301.875
22	AVJL	31	299.8236
23	RIMR	31	331.875
24	PVR	30	253.0756
25	AVJR	29	302.4691
26	ADEL	29	438.9381
27	RIML	29	322.8739
28	RMGL	28	320
29	RIS	28	303.75
30	PVT	28	301.7595
31	AVL	27	300.9375
32	DVC	27	350.6151
33	PVNR	26	2100.4405
34	RIGR	25	324.375
35	RICL	25	419.6481
36	PVPR	25	310.3125
37	ADAL	25	438.9381
38	CEPDR	24	396.3093
39	AVKR	24	303.75
40	AVHL	24	347.4427
41	ADER	24	440.2116
42	AIZR	24	438.8007
43	AIZL	24	443.1858
44	RMDVR	24	310.1504
45	RICR	24	429.375
46	PVQR	24	328.7478
47	PVNL	24	2100.4405
48	HSNL	24	420.531
49	RMGR	23	317.4603
50	RMDL	23	314.1509
51	PVPL	23	306.1584
52	AVHR	22	342.1517
53	AVG	22	309.375
54	AVFL	22	1676.1905
55	ASHR	22	315
56	VC03	22	1612.6984
57	SMDDR	22	318.75
58	ADAR	22	438.8007
59	RMDR	22	313.2275
60	RIR	22	309.375
61	ADLL	21	303.7736
